# Exploring the role of CITED transcriptional regulators in the control of macrophage polarization

**DOI:** 10.3389/fimmu.2024.1365718

**Published:** 2024-04-05

**Authors:** Derek A. Wiggins, Jack N. Maxwell, David E. Nelson

**Affiliations:** Department of Biology, Middle Tennessee State University, Murfreesboro, TN, United States

**Keywords:** CITED2, CITED1, STAT1, macrophage, innate immunity, inflammation, NF-κB, CBP/p300

## Abstract

Macrophages are tissue resident innate phagocytic cells that take on contrasting phenotypes, or polarization states, in response to the changing combination of microbial and cytokine signals at sites of infection. During the opening stages of an infection, macrophages adopt the proinflammatory, highly antimicrobial M1 state, later shifting to an anti-inflammatory, pro-tissue repair M2 state as the infection resolves. The changes in gene expression underlying these transitions are primarily governed by nuclear factor kappaB (NF-κB), Janus kinase (JAK)/signal transducer and activation of transcription (STAT), and hypoxia-inducible factor 1 (HIF1) transcription factors, the activity of which must be carefully controlled to ensure an effective yet spatially and temporally restricted inflammatory response. While much of this control is provided by pathway-specific feedback loops, recent work has shown that the transcriptional co-regulators of the CBP/p300-interacting transactivator with glutamic acid/aspartic acid-rich carboxy-terminal domain (CITED) family serve as common controllers for these pathways. In this review, we describe how CITED proteins regulate polarization-associated gene expression changes by controlling the ability of transcription factors to form chromatin complexes with the histone acetyltransferase, CBP/p300. We will also cover how differences in the interactions between CITED1 and 2 with CBP/p300 drive their contrasting effects on pro-inflammatory gene expression.

## Introduction

Macrophages are innate immune cells that exhibit remarkable phenotypic plasticity, adopting contrasting polarization states over the course of an infection to best contribute to tissue defense and repair (1). During an infection’s opening stages, exposure to interferon-γ (IFNγ) and/or pathogen-associated molecular patterns (PAMPs), such as lipopolysaccharide (LPS), promotes M1 polarization of naïve (M0) macrophages (2). In this state, macrophages secrete pro-inflammatory cytokines and exhibit heightened antimicrobial activity, partially driven by inducible nitric oxide synthase (iNOS) expression, which produces nitric oxide (NO) as a precursor for microbicidal reactive nitrogen species (RNS) (3–5). As an infection resolves, rising levels of interleukin (IL)-4 and IL-13 repolarize macrophages to the M2 state, which is associated with tissue repair and anti-inflammatory cytokine secretion. Additionally, the production of cytotoxic RNS is attenuated through arginase-1 (Arg1) expression, which competes with iNOS for arginine, the common substrate of both enzymes (6).

The transition between polarization states involves broad transcriptional reprogramming affecting >1,000 genes (7, 8). The molecular mechanisms governing these changes are of particular importance to human health as the failure to downregulate pro-inflammatory gene expression programs or activate them results in adverse outcomes. Specifically, inappropriate M1 activation contributes to inflammatory disorders, such as atherosclerosis, asthma, diet-induced obesity, and insulin resistance (9–13). Conversely, intracellular pathogens that colonize macrophages, including *Toxoplasma gondii* and *Salmonella enterica* serovar Typhimurium, utilize effectors that target these same mechanisms to block M1 or promote M2 polarization of host cells, creating a more permissive environment for growth and persistence (14–19).

While multiple transcription factors, including Krüppel-like factors (KLFs), hypoxia-inducible factor 1 (HIF1), and p53 (20–27) contribute to gene expression changes during polarization, the nuclear factor kappaB (NF-κB) and Janus kinase (JAK)/signal transducer and activation of transcription (STAT):interferon regulated factor (IRF) pathways are considered the primary controllers (28–30). Each is regulated by their own pathway-specific feedbacks that amplify or attenuate the response to polarizing stimuli. These include reciprocal positive feedback loops between *Stat1* and *Irf1* (31), and the various negative feedbacks (i.e., inhibitor-kappaB (IκB)α, ϵ, and A20) that prevent premature or prolonged NF-κB activity (32–39). However, these are supplemented by feedbacks that impact the activity of multiple polarization regulatory pathways, providing a higher level of control and coordination (40). For example, suppressor of cytokine signaling 1 (SOCS1) attenuates STAT1 activity by inhibiting JAKs and blocking NF-κB activity through recruitment to specific NF-κB-regulated gene promoters, stimulating nuclear p65 (RelA) NF-κB protein degradation (41–43). In this review, we will bring together recent findings showing how the CBP/p300-interacting transactivator with glutamic acid/aspartic acid-rich carboxy-terminal domain (CITED) family of transcriptional co-regulators contribute to this broader level of control. Specifically, we will describe how CITED1 and 2 proteins operate as common regulators of NF-κB, HIF1, IRF1, and the STAT family of transcription factors and explore how the antagonistic activities of these proteins shape the transcriptional response of macrophages to polarizing signals.

## The CITED family of transcriptional co-regulators

Originally named melanocyte-specific gene 1 (*msg1*) following its discovery in highly pigmented B16-F1 murine melanoma cells, *Cited1* was the first member of the *Cited* gene family to be described (44), with the same study also identifying *Cited2*, initially dubbed msg-related gene 1 (*mrg1*) based on sequence homology. This was quickly followed by *Cited3*, present in avian, amphibian, and fish species (45, 46) but absent in mice and humans, and *Cited4*, the only other family member expressed in mammalian systems (47, 48). These genes participate in different and diverse biological processes, such as melanocyte pigmentation (44), placentation (49), embryonic development (50), and differentiation (48). They are even implicated in different sets of pathological conditions, including congenital heart disease and cancer (51–64). In all of these biological processes, they function as transcriptional co-regulators (48, 65, 66).

**Figure 1:**
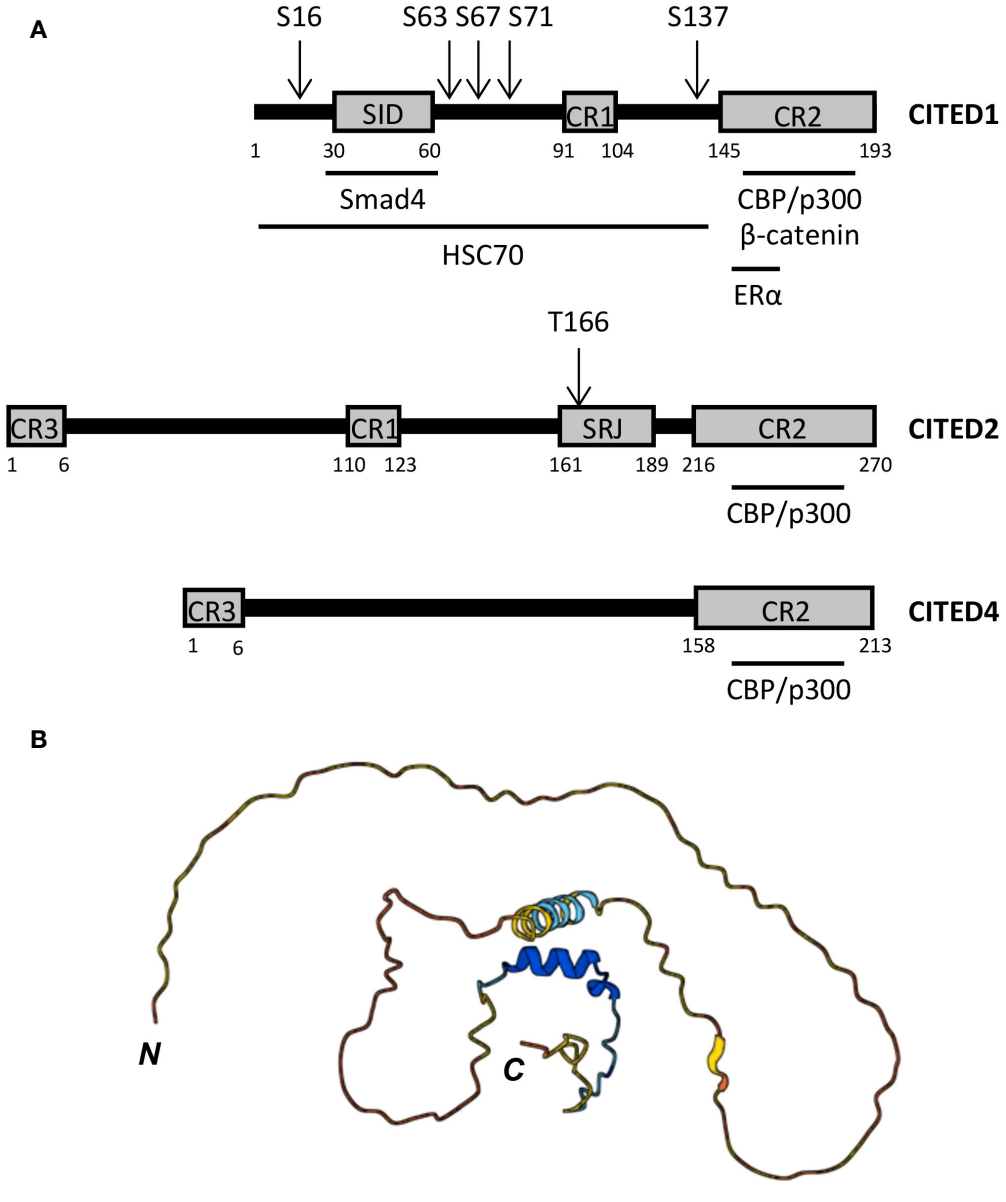
*CITED protein structure*. **(A)** Domain structure of human CITED1, 2, and 4. CR, conserved region; SID, Smad-interacting domain; SRJ, serine-rich junction. Black horizontal bars below each peptide map mark the regions required for the indicated protein:protein interactions, including the minimal CBP/p300-binding region, which is entirely contained within the CR2 domain of each CITED protein. Known phosphorylation sites are marked by arrows above each peptide map. **(B)** Predicted structure of human CITED1 generated in AlphaFold (67, 68). N- and C-termini are marked. Per-residue model confidence scores (pLDDT) on a 1-100 scale are encoded as follows: Dark blue, very high (pLDDT > 90); light blue, high (90 > pLDDT > 70); yellow, low (70 > pLDDT > 50); orange, very low (pLDDT < 50). The prediction indicates that CITED1 is largely unstructured.

As transcriptional co-regulators, CITED proteins lack DNA binding domains and are not known to form dimers or multimers. Instead, these proteins contain sets of conserved region (CR) motifs including the centrally located CR1 motif (~14 aa) present in CITED1, 2, and 3, a shorter CR3 motif (~6 aa) at the N-terminus of CITED2, 3, and 4, and the longer C-terminal CR2 domain (48-55 aa) common to all CITED proteins (**Figure 1A**) (47, 65). Outside these regions, CITED proteins exhibit minimal sequence similarity and are largely unstructured (**Figure 1B**), but can contain protein interaction motifs, such as the Smad-interacting domain (SID) identified in CITED1 (65).

**Figure 2:**
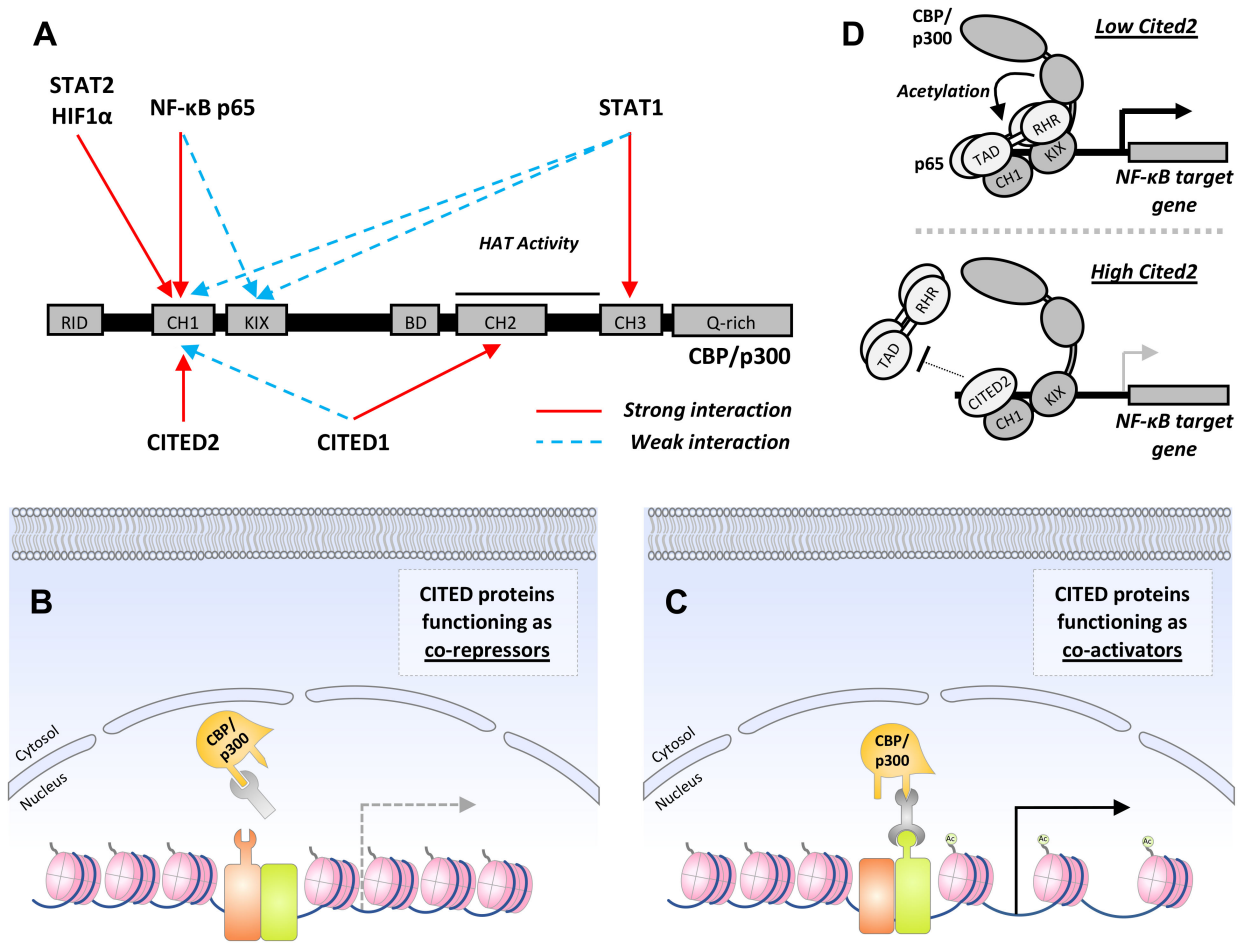
*CITED proteins function as co-activators or repressors by regulating CBP/p300:transcription factor complex formation.* **(A)** Schematic showing domains common to CBP and p300 with the interaction sites utilized by transcription factors that regulate macrophage M1 polarization, and the CITED transcriptional co-regulators are marked above and below, respectively. Strong and weak interactions are encoded in red and blue, respectively. RID, receptor-interacting domain; CH1-3, cysteine and histidine-rich regions; KIX, CREB-binding domain; BD, bromodomain; Q-rich, glutamine-rich domain. In some cases, interactions between a transcription factor and CITED protein with a common CH domain are known to be mutually exclusive (i.e., CITED2 and HIF1α with CH1). (B+C) General mechanisms for CITED protein co-repressor and co-activator activity. **(B)** By associating with the same docking site used by a transcription factor, CITED proteins can operate as co-repressors through a simple competitive inhibition mechanism. This is the mechanism by which CITED2 suppresses HIF1α-dependent gene expression. **(C)** Alternatively, CITED proteins can facilitate CBP/p300:transcription factor complex formation by operating as co-activators or adaptors. This results in the recruitment of CBP/p300 to *cis*-regulatory sites, histone acetylation, and chromatin remodeling that favors gene expression. Examples of this include CITED1:Smad4 and CITED1:STAT3:ERα complex formation Ac represents histone acetylation. **(D)** A specific example of CITED2 co-repressor activity: The p65 subunit of canonical NF-κB transcription factors makes bipartite interactions with CBP/p300, contacting both the CH1 and KIX domain, recruiting CBP/p300 to κB sites in target gene promoters. By disrupting the stronger of the two interactions, which occurs between the p65-TA2 subdomain and CBP/p300 CH1 domain, CITED2 inhibits CBP/p300-mediated acetylation of p65 itself, reducing NF-κB DNA-binding activity, and destabilizing the CBP/p300: NF-κB chromatin complex, thereby reduced expression of pro-inflammatory NF-κB target genes.

While no clear function has been ascribed to CR1 and 3, the CR2 domain is essential for transcriptional co-regulatory activity (65, 69). Analogous to the transactivation domains (TADs) of STAT, NF-κB, and HIF1 proteins, CR2 enables CITED proteins to associate with one or more of the three cysteine-histidine-rich (CH) domains within the histone acetyltransferase (HAT), CREB binding protein (CBP) and its paralogue, p300 (65, 70–72) (**Figure 2A**). In this way, CITED proteins can inhibit or facilitate the formation of transcription factor:CBP/p300 complexes at gene cis-regulatory sites by blocking access to specific CH domains or functioning as a co-factor for transcription factors that weakly associate with CBP/p300 (66, 73–76) (**Figures 2B, C**).

## CITED2 interactions with CBP/p300

The first indication that CITED proteins function as transcriptional co-repressors was provided by Bhattacharya et al. (77). In this study, p35srj, a CITED2 isoform containing a 39 aa serine-glycine-rich junction (SRJ) between CR1 and 2 (**Figure 1A**), was identified in a screen for proteins interacting with p300-CH1 (TAZ1). Endogenous p35srj was shown to be constitutively nuclear in U2OS human osteosarcoma cells with almost the entire cellular pool found in CBP/p300 complexes, and the minimal binding region necessary for p300-CH1 interaction completely contained within the CR2 domain (224-255 aa). This study also showed that p35srj could inhibit HIF1-dependent gene transactivation by competing with the C-terminal TAD (CAD) of HIF1α for CH1 binding (**Figures 2A, B**). This is consistent with subsequent work showing synthetic membrane-permeable peptides, based on the CITED2 CR2 domain, inhibit HIF1α:p300 complex formation and HIF1-dependent gene expression (73).

One of the more important outcomes of the Bhattacharya study was the demonstration that CITED2 preferentially binds the CH1 domain of CBP/p300, distinguishing it from CITED1, which primarily associates with CH2 (65) (**Figure 2A**). Specifically, Bhattacharya et al. showed that p35srj did not reduce the activity of transcriptional regulators binding p300 outside the CH1 domain, including SREBP2 and src-1, which associate with the CREB-binding domain (KIX) and glutamine-rich domains of CBP/p300, respectively (78, 79). This result was supported by subsequent studies showing that CITED2 blocks the binding of the HIF1α N-terminal TAD (NAD) to p300-CH1 but not CH3 (74). More recently, NMR spectroscopy was used to identify the specific CH1 binding surface utilized by CITED2 and it was found that while the CITED2 CR2 domain and the HIF1α CAD bind to CH1 with similar affinity, they occupy distinct but overlapping binding positions (70). Collectively, these data indicate that CITED2 proteins may function as general inhibitors of transcription factors that use the CH1 domain for docking with CBP/p300.

## CITED2 as a modulator of macrophage pro-inflammatory gene expression

### Regulation of HIF1α activity

While HIF1α signaling is most closely associated with the hypoxia response, it also plays a significant role in macrophage polarization under normoxic conditions. Induced by M1 polarizing stimuli, including LPS (23, 26), HIF1α modulates the expression of almost half of all IFNγ-responsive genes (80). It also provides a protective role during *Leishmaina donovani* and *Mycobacterium tuberculosis* infection (81, 82) by regulating the shift to aerobic glycolysis and increasing macrophage microbicidal activity (80, 83, 84).

In 2018, the Mahabeleshwar lab demonstrated that CITED2 is present in human and murine macrophages and functions as a general inhibitor of pro-inflammatory gene expression and macrophage activation (85). This was found to be dependent, in part, on the ability of CITED2 to suppress HIF1α-regulated gene expression, demonstrated using CITED2-deficient bone marrow-derived macrophages (BMDMs). While this study did not discount the competitive inhibition model (**Figure 2B**), whereby CITED2 blocks HIF1α:CBP/p300 complex formation (74, 77), it showed that CITED2 enhances the expression of Egln3, a HIF1α negative regulator, in turn destabilizing the transcription factor (86). Furthermore, this study also showed that CITED2 expression is dynamic and is temporarily down-regulated in LPS or IFNγ stimulated BMDMs, presumably to disinhibit or permit expression of M1-associated genes after appropriate signals have been encountered.

### Regulation of NF-κB activity

Canonical p65-containing NF-κB transcription factors play a central role in regulating M1-associated gene expression. Activated by cytokines (e.g., TNFα) and PAMPs, NF-κB regulates genes associated with cell survival, proliferation, and inflammation (87). Many of these genes are co-regulated by HIF1α and STAT1, including a principal M1 marker, *Nos2*, which exhibits synergistic expression following NF-κB and STAT1 activation (88). While the signaling cascades that trigger NF-κB activity vary between stimuli, most converge at the level of the inhibitor-kappaB kinase (IKK) signalosome (89). Once activated, the IKK signalosome phosphorylates the IκB proteins that keep dimeric NF-κB transcription factors locked in an inactive state in the cytoplasm (90), marking them for destruction by the proteasome (91, 92). This allows NF-κB proteins to translocate to the nucleus, and transactivate NF-κB-responsive gene promoters (93).

Like HIF1α, NF-κB also recruits CBP/p300 to target gene *cis*-regulatory sites to initiate gene transcription and does so in a bipartite fashion (94, 95). The TA2 subdomain of the p65 C-terminal TAD interacts strongly with the CBP/p300 CH1 domain, whereas the N-terminal Rel homology region, when phosphorylated at Ser276, forms a weaker interaction with the KIX domain (**Figure 2A**). The p65 TA2 subdomain is intrinsically disordered, and it associates with interlinked hydrophobic grooves on the surface of CH1 (95). Experiments using p65 TA2 and Ser276 mutants, ablating KIX or CH1 binding activity, respectively, show that these interactions are functionally separable and regulate different NF-κB target gene sets. Mutation of the TA2 domain affects a broader set of genes, including *Ptgs2* and *Cxcl1*, than the loss of Ser276 phosphorylation, suggestive of the important role played by the p65-TA2:CBP-CH1 interaction. Additionally, expression of the main NF-κB negative regulators, IκBα and A20, encoded by *Nfkbia* and *Tnfaip3*, required both interactions (95).

Given the importance of the p65-TA2:CBP-CH1 interaction for target gene transactivation, it is unsurprising that CITED2 operates as a negative regulator of macrophage NF-κB signaling (96, 97). This was first shown by Lou et al, demonstrating that CITED2 attenuates NF-κB target gene expression in J774 macrophage-like cells and BMDMs (97). Their data was consistent with CITED2 operating as a competitive inhibitor of p65:CBP/p300 interactions, as i) CITED2 expression had no effect on up-stream processes, including IκBα phosphorylation and degradation, and ii) mutations within the CITED2 CR2 domain ablating p300 binding resulted in the complete loss of CITED2 co-repressor activity. Lou et al. also showed that CITED2 expression reduced p300-dependent acetylation of p65. This likely explains the reduction in p65 and RNA polymerase II detected at *Tnfaip3* and *Cxcl8* promoters by ChIP as acetylation of p65 at Lys310 is required for DNA binding (98). These findings are largely corroborated by Pong et al, which used complementary gain- and loss-of-function manipulations coupled with RNA sequencing and gene set enrichment analysis (GSEA) to investigate the role of CITED2 in a mouse model of lung inflammation (96). BMDMs derived from *Cited2^fl/fl^:Lyz2^cre^* mice exhibited enhanced expression of multiple pro-inflammatory NF-κB targets following LPS stimulation, including *Il1β*, *Cxcl2*, *Tnf*, and *Klf6*. In agreement with Lou et al, the investigators found that CITED2 inhibited LPS-induced p65-promoter binding without affecting the release of NF-κB from IκB complexes. Additionally, Pong et al. showed that loss of myeloid CITED2 expression significantly increased macrophage recruitment to lung tissue following intratracheal delivery of zymosan in a murine model of acute lung inflammation. Collectively, these studies support the notion that CITED2 functions as an NF-κB co-repressor by preventing productive p65:CBP/p300 interactions, reducing p65 acetylation and DNA binding activity. This hampers NF-κB:CBP/p300 complex recruitment to κB sites and attenuates pro-inflammatory gene expression (**Figure 2D**).

### Regulation of JAK-STAT activity

As an additional finding of the Pong et al. study, data from their GSEA analysis indicated that CITED2 loss enhanced expression of IFNγ-responsive genes in LPS-stimulated BMDMs, raising the possibility that CITED2 also operates as a suppressor of STAT1-IRF1 signaling. This was explored by the Mahabeleshwar lab in Zahar et al. using the same *Cited2^fl/fl^:Lyz2^cre^* mouse model (99). Here, they showed that CITED2 loss enhanced expression of both STAT1 and IRF1 gene targets in IFNγ-stimulated BMDMs, including members of the *Isg*, *Ifit*, and *Oasl* gene families. As observed for NF-κB, the effect of CITED2 expression on STAT1 signaling was confined to the nucleus as CITED2 loss had no effect on STAT1 protein levels or phosphorylation at Tyr701 following IFNγ stimulation. Rather, CITED2 deficient BMDMs showed increased STAT1-chromatin binding. Most significantly, these cells showed enrichment of STAT1 at the *Irf1* promoter, correlating with increased transcription of the *Irf1* gene and heightened IRF1 protein levels. This supports a model where CITED2 is interrupting the positive feedback between STAT1 and IRF1, thereby attenuating the STAT1-IRF1 transcriptional program that is crucial for macrophage M1 polarization.

Quite how CITED2 inhibits STAT1 activity has not been fully resolved and is likely to be distinct from the HIF1 and NF-κB competitive inhibition mechanisms due to differences in how STAT1 interacts with CBP/p300. While there are some variations in how this interaction is reported (100–102), there appears to be two contact points between these proteins involving the STAT1 N- and C-termini. The N-terminus, which plays a role in cooperative binding interactions with DNA (103), interacts with the CBP/p300 KIX domain (104), whereas the C-terminal TAD binds both CH1 and CH3 (72). However, it exhibits a strong preference for CH3, with the CH1 binding interaction being 100-fold weaker. Additionally, Zafar et al. show that STAT1 and CITED2 are both recruited to the IRF1 promoter in IFNγ-stimulated BMDMs (99). Taken together, these data bring a CITED2:STAT1 competitive inhibition model into question.

Interestingly, STAT2 proteins, which have a slightly longer C-terminal TAD than STAT1, preferentially bind CH1, interacting with the same surfaces as HIF1α and CITED2 (72, 105). This indicates that CITED2 may also function as a STAT2 co-repressor following type I interferon stimulation, which is consistent with the Pong et al. transcriptome data showing enhancement of the IFNα-response gene sets in LPS-stimulated BMDMs from *Cited2^fl/fl^:Lyz2^cre^* mice (96). As a small caveat, STAT2 is known to recruit other HATs, such as GCN5 rather than CBP/p300, to some ISG enhancers (106), which may limit the impact of CITED2 on STAT2 targets.

The interplay between CITED2 and the JAK/STAT signaling pathway is not restricted to STAT1 and STAT2. Clear genetic and protein interactions with other STAT family members that contribute to the shaping of the M1 and M2 transcriptome have also been demonstrated. Within the past year new data generated using the *Cited2^fl/fl^:Lyz2^cre^* mouse model has identified a relationship between CITED2, STAT3, and STAT5 that modulates the macrophage gene expression programs and contributes to the inflammation and insulin resistance observed during diet-induced obesity (107). In this system, CITED2 was found to suppress STAT3 and STAT5 target gene expression in BMDMs. For STAT5, this was shown to involve the suppression of LPS-induced Tyr694 phosphorylation, a modification necessary for STAT5a nuclear accumulation, dimerization, and activity. An important downstream effect of this was increased expression of B cell lymphoma 6 (BCL6), a transcriptional repressor that restrains the macrophage pro-inflammatory response and is itself inhibited at the transcriptional level by STAT5 (108, 109). In effect, this CITED2-STAT5-BCL6 axis enables CITED2 to control broad swaths of the M1 transcriptional program in a manner that is at least partially separable from its own role as a co-repressor of transcription factor:CBP/p300 interactions.

In addition to acting as a general inhibitor of M1-associated pro-inflammatory gene expression, the relationship between STAT6 and CITED2 enables it to make meaningful contributions to the M2 transcriptome. Just as CITED2 expression is inhibited by M1-polarizaing signals, an M2-polarizing environment (IL-4 or IL-13 stimulation) promotes a modest increase in CITED2 expression, which was found to be regulated at a transcriptional level by STAT6 (85). Under these conditions, CITED2 proteins form complexes with peroxisome proliferator-activated receptor gamma (PPARγ), a nuclear receptor that collaborates with STAT6 to regulate IL-4-stimulated anti-inflammatory gene expression (110, 111). Not only was CITED2 shown to enhance PPARγ-dependent gene expression in macrophages, but it was also found to stabilize the interaction between PPARγ and the *Arg1* enhancer in IL-4-stimulated BMDMs and was associated with increased expression on this M2 marker. Interestingly, IL-4 has also been shown to promote the STAT6-dependent transcription of *Cited1* in human adipocytes with CITED1 potentially functioning as a thermogenic protein in this context (112). Whether it is similarly regulated in myeloid cells has yet to be investigated.

Collectively, these studies show that the contrasting regulation of CITED2 expression under M1 or M2-stimulating conditions creates an elegant switching mechanism that ensures clear transitions between polarization states. While this may primarily utilize the canonical transcriptional co-repressor activities of CITED2, its ability to influence HIF1α and BCL6 levels, as well as facilitating PPARγ-promoter interactions, extends the influence of CITED2 over a wider set of pro- and anti-inflammatory genes.

## Cited1, a newly discovered regulator of macrophage pro-inflammatory gene expression

While *Cited2* expression is ubiquitous, *Cited1* is largely restricted to a small number of tissues (113), and was thought to be absent in macrophages (85). This perception was changed in 2020 when *Cited1* transcripts were detected in M1-polarized RAW264.7 murine macrophage-like cells infected with the fungal pathogen, *Cryptococcus neoformans* (114). It was subsequently determined that exposure of these cells to IFNγ for periods greater than 24 hours stimulated expression of CITED1 proteins, regulated by both STAT1 and IRF1 at the transcriptional level. *Cited1* expression was unaffected by other M1 polarizing stimuli (i.e., LPS), indicating that it operates as an interferon-stimulated gene (ISG) (113). This pattern of regulation is in stark contrast to *Cited2*, which is transiently downregulated following short periods of exposure (6 hours) to M1 polarizing stimuli (LPS or IFNγ) and upregulated by M2 stimuli (IL-4 or IL-13) (85), indicating that the circumstances under which CITED1 and 2 operate may be minimal or non-overlapping.

A further regulatory difference between CITED1 and 2 is their subcellular localization under varying polarizing conditions. While CITED2 has been shown to be constitutively nuclear (97), CITED1 is predominantly cytoplasmic in unstimulated cells but becomes enriched in the nucleus following IFNγ stimulation (113). Quite how this shift in subcellular distribution is controlled is presently unclear, but it may be connected to the increased phosphorylation of the protein observed in IFNγ-stimulated macrophages as CITED1 phosphorylation at Ser79 following parathyroid-hormone-stimulation in murine osteoblastic cells is known to be associated with its nuclear translocation (115, 116).

The differences between CITED1 and 2 go beyond the regulation of their expression and localization and extends to their function with the two transcriptional regulators exerting opposing effects on macrophage pro-inflammatory gene expression. This was shown by Subramani et al. using a complementary loss- and gain-of-function approach, where it was found that CITED1 enhances STAT1- and IRF1-regulated gene expression in IFNγ-stimulated macrophages, increasing transcript levels of *Ccl*, *Isg*, and *Ifit* family members amongst many others (113). These data suggest that CITED1 is functioning as a transcriptional co-enhancer (**Figure 2C**), versus the largely co-inhibitory effects of CITED2 in the same context. This is perhaps unsurprising as (i) neither STAT1 or IRF1 bind CBP/p300 via CH2, which is thought to be the preferred CITED1 interaction site (72, 117), and (ii) CITED1 has been shown to function as a co-enhancer in other contexts (65, 69, 118). For example, CITED1 enhances Smad4-dependent gene expression by stabilizing CBP/p300:Smad4 complexes, interacting with both proteins (65). Similarly, CITED1 acts as a co-factor for the assembly of STAT3:estrogen receptor-α (ERα) transcriptional complexes in neurons as part of leptin signaling, contributing to whole-body energy homeostasis (118). This is of relevance as STAT3 contributes to IFNγ-stimulated gene expression in macrophages (119, 120). It also illustrates the close relationship that both CITED1 and 2 share with the STAT family of transcriptional regulators.

## Concluding remarks and future directions

CBP/p300 serves as a convergence point for the principal signaling pathways that control macrophage polarization, with their associated transcription factors either competing for shared CBP/p300 binding surfaces or acting cooperatively to recruit it to enhancers. The CITED family of transcriptional regulators largely operate at this level and their differing binding preference for the three CH domains within CBP/p300 enable them to hinder or facilitate this process, shaping macrophage pro- and anti-inflammatory gene expression and phenotype. This process is assisted by the contrasting regulation of *Cited1* and *2*, which results in the highest expression of CITED2 in the absence of M1 or presence of M2-polarizing stimuli, dampening pro-inflammatory gene expression. Whereas CITED1 is only expressed at detectable levels in cells exposed to IFNγ for extended periods, enhancing ISG expression and maintaining the pro-inflammatory response.

The contributions of CITED2 to macrophage polarization and its role in a variety of inflammatory disorders, including atherosclerosis and diet-induced obesity, has been well explored using mouse models and primary cells (85, 96, 97, 99, 107). Additionally, the molecular interactions of this protein with CBP/p300 are well understood to the extent where the CITED2 CR2 domain has been used as the basis for cell-permeable peptide inhibitors of HIF1:CBP/p300 interaction with clinical potential (73). Far less is known about CITED1 in this context and our current knowledge is based exclusively on studies in murine macrophage cell lines (113, 114). Those *in vivo* studies that have been performed have instead focused on the impact of *Cited1* gene deletion or the effect of CITED1 mutants on development and cancer rather than its role in myeloid cells (60, 121). Perhaps most importantly, the CITED1:CBP/p300 interaction has not been fully resolved with the proposed CH2-binding preference of CITED1 based only on qualitative *in vitro* pull-down mapping experiments using large GST-fused portions of p300 containing more than just the CH domains (65). While the CR2 domains of CITED1 and 2 are highly similar, (82% amino acid identity based on aa 161-209 of human CITED2 (44);), there are notable differences, including a 7 aa insert at the end of the domain that is absent from CITED1, indicating that a difference in CH binding preference is at least plausible. Future in-depth structural studies will provide clarity in this area. Should these differences in binding preference prove to be valid then their contributions to the seemingly opposite effects of CITED1 and 2 on IFNγ-stimulated gene expression could be explored using CITED mutants where the CR2 domains have been exchanged. Hypothetically, this would convert CITED1 from a transcriptional co-enhancer to a co-repressor.

A further unresolved question concerns the potentially differing relationships between the CITED proteins and the HATs, CBP and p300. It is currently unclear whether they exhibit a binding preference for one or the other, which is potentially significant as while the activities of CBP and p300 are at least partially overlapping, they are known to have separate functions and regulate distinct collections of genes (102). This is relevant within the context of macrophage polarization as CBP and p300 interact differently with STAT1. As an example of this, CBP, but not p300, has been shown to directly acetylate the STAT1 DNA binding domain at Lys410 and 413 (122). This has two important consequences; i) acetylated STAT1 binds nuclear NF-κB dimers, reducing their DNA-binding and transcriptional regulatory activity, and ii) signals for the nuclear export and dephosphorylation of STAT1 as part of a phosphorylation-acetylation switch that deactivates the protein (123). This would suggest that by interfering with the STAT1:CBP interaction, CITED proteins could, perhaps counterintuitively, extend STAT1 nuclear occupancy and enhance NF-κB activity.

As a similar concern, our understanding of how post-translational modifications (PTMs) of the CITED proteins themselves impact their function and activity remains incomplete. Most of those reported to date are not known to occur within myeloid cells and include the MAPK1-dependent phosphorylation of CITED2 at T166 at the N-terminal end of the SRJ domain (124), and the cell cycle-regulated phosphorylation of CITED1 mapped to five serine residues throughout the protein (125), with these modifications altering the coactivator functions of the proteins by influencing CBP/p300 binding. So far only CITED1 has been shown to be phosphorylated in macrophages in response to polarizing signals (in this case, IFNγ), but the modification sites, kinases responsible, and the functional consequences have yet to be elucidated. Additionally, just as many of the transcription factors that interact with CBP/p300 are acetylated by these HATs as an important part of their regulation [e.g., STAT1 and p65 NF-κB (98, 122, 123)], it seems plausible that the CITED proteins will also be acetylation targets or subject to other PTMs.

In closing, much remains to be discovered about how CITED proteins contribute to the control of macrophage polarization. Continued efforts in this area will help us better understand the various CBP/p300-dependent and independent mechanisms used by these proteins to tune pro- and anti-inflammatory gene expression and how the CITED proteins themselves are regulated.

## Author contributions

DW: Writing – original draft, Writing – review & editing. JM: Writing – original draft, Writing – review & editing. DN: Writing – original draft, Writing – review & editing, Conceptualization, Funding acquisition, Supervision.
